# Improving Non-Clinical Engagement in Support for Patients with Dual-Diagnosis Within an Assertive Outreach Team in York

**DOI:** 10.1192/bjo.2026.11286

**Published:** 2026-06-30

**Authors:** Thomas Riley, Christiana Elisha-Aboh

**Affiliations:** Tees, Esk and Wear Valleys NHS Foundation Trust, York, United Kingdom

## Abstract

**Aims:**

Assertive Outreach Teams (AOT) provide enhanced community support for people with serious mental illness and complex needs, amongst whom there is a higher risk of comorbid alcohol use, for which engagement in structured interventions can be challenging. Mental health professionals can deliver ‘Bitesized’ interventions directly to patients, however this can represent a relatively infrequent window of opportunity. AOT patients often have regular contact with care and support staff who may not have clinical expertise. Empowering this wider support network to discuss alcohol use with patients could reduce alcohol-related harm.

**Methods:**

A teaching session was delivered to non-clinical staff at a residential home which supports patients under the care of the AOT. The session covered background knowledgeregarding alcohol use and health, and how the staff could deliver informal ‘Bitesized’ interventions through their daily interactions with residents. Pre- and post-session feedback was obtained through questionnaires which utilised Likert scales to assess confidence in discussing key topics and the likelihood they would talk to patients about alcohol use. The questionnaire also assessed specific knowledge markers including safer alcohol limits and risk levels.

**Results:**

8 questionnaires were returned.

Post-session, 100% of respondents reported confidence in discussing alcohol use with residents, including a 33% increase in those feeling ‘very confident’. The same results were found when asked about discussing relevant physical health risks. In the pre-session questionnaire, high-risk drinking levels were correctly identified by 50% of respondents, increasing to 88% post-session.

When asked pre-session about confidence discussing mental health risks relating to alcohol use, 25% of respondents selected ‘very confident’ and 13% selected ‘not confident’. Post-session, 100% reported confidence, with 71% selecting ‘very confident’.

Following the session, 86% of respondents said they would talk to residents more frequently about alcohol use, with 100% of respondents reporting the session to be helpful.

**Conclusion:**

Results suggest that brief teaching delivered to non-clinical staff can improve confidence in discussing alcohol use, and related risks, with mental health patients, and increase the likelihood they will have these discussions more frequently. This project offers only a small sample and does not provide information about whether the results will translate into meaningful change in patient outcomes. It offers a platform for ongoing rehabilitationwork and demonstrates the utility in multidisciplinary working in delivering holistic care for patients with complex mental health needs. Future directions may include trialling similar or substance-related interventions elsewhere and designing sessions for delivery directly to patients.

